# The Effect of Attention Deficit/Hyperactivity Disorder on Physical Health Outcomes: A 2-Sample Mendelian Randomization Study

**DOI:** 10.1093/aje/kwaa273

**Published:** 2020-12-16

**Authors:** Beate Leppert, Lucy Riglin, Robyn E Wootton, Christina Dardani, Ajay Thapar, James R Staley, Kate Tilling, George Davey Smith, Anita Thapar, Evie Stergiakouli

**Keywords:** ADHD, childhood obesity, coronary artery disease, Mendelian randomization

## Abstract

Attention-deficit/hyperactivity disorder (ADHD) is associated with a broad range of physical health problems. Using different research designs to test whether ADHD has a causal role in these associations is important because comorbid health problems increase the serious social and economic impacts of ADHD. We used 2-sample Mendelian randomization (MR) to infer causal relationships between ADHD and previously implicated physical health conditions. Different MR methods were used to test the robustness and plausibility of our findings. Consistent findings underwent bidirectional and multivariable MR. We found evidence of ADHD having a causal effect on childhood obesity (odds ratio = 1.29, 95% confidence interval: 1.02, 1.63) and coronary artery disease (odds ratio = 1.11, 95% confidence interval: 1.03, 1.19) with consistent results across MR approaches. There was additional MR evidence for a bidirectional relationship between ADHD and childhood obesity. The relationship with coronary artery disease attenuated when controlling for childhood obesity. There was little evidence for inferring a causal effect on other cardiometabolic, autoimmune, allergic, and neurological diseases. Our findings strengthen the argument for effective treatment of children with ADHD, and suggest that clinicians who manage ADHD need to be aware of the risk of childhood obesity to reduce future risks of coronary artery disease.

## Abbreviations

ADHD: attention deficit hyperactivity disorder
BMI: body mass index
CAD: coronary artery disease
CI: confidence interval
GWAS: genome-wide association study
IVW: inverse variance weighted
MR: Mendelian randomization
MVMR: multivariable Mendelian randomization
SNP: single-nucleotide polymorphism

Attention-deficit/hyperactivity disorder (ADHD) typically begins in early childhood and has a worldwide prevalence of approximately 5% in school-aged children (1, 2). Approximately 65% of children diagnosed with ADHD have symptoms and impairment that persist into adulthood (3), and ADHD can lead to educational, social, and occupational difficulties (4). The economic impact of ADHD is substantial, with the annual national excess costs for ADHD ranging from $143 billion to $266 billion in the United States; most of these costs are incurred in adulthood (5). A proportion of these costs is due to higher mortality and morbidity rates, although the reasons behind these associations are currently unclear (6, 7).

There is growing evidence from case-control and cohort studies that ADHD is associated with a broad range of physical health problems. These include obesity (8), type 2 diabetes mellitus, and hypertension (6)—known risk factors for cardiovascular disease. ADHD is associated with asthma (9); allergic rhinitis (10); and autoimmune conditions, including psoriasis and rheumatoid arthritis (11, 12); as well as childhood epilepsy (13) and migraine (14). Some of these reported associations withstand meta-analyses. However, conventional observational studies are problematic, because associations can arise due to selection bias, reverse causation, and residual confounding (15).

Alternative research designs are needed to infer causation; Mendelian randomization (MR) offers an approach by reducing bias from confounding and reverse causation (16). The rationale behind MR is that genetic variants that are robustly associated with an “exposure” (ADHD in this instance) can be used as instrumental variables for that exposure. Theoretically, they are unconfounded indicators because they are determined randomly at conception and segregate to viable offspring independently of environmental influences (17, 18). Thus, provided certain assumptions are met, MR is akin to a randomized controlled trial where the intervention increases the likelihood of ADHD. In this study, we used a 2-sample MR design to estimate causal effects of ADHD (indexed by genetic instruments) on metabolic, cardiovascular, autoimmune, allergic, and neurological conditions. These conditions were selected if 1) they had been associated previously with ADHD through observational studies and 2) had publicly available summary statistics from large-scale genome-wide association studies (GWASs). When findings suggested a potentially causal effect, we further tested for possible bidirectional effects. ADHD typically arises early in development and thus precedes the onset of most physical health conditions in the children. However, bidirectional analyses could potentially detect dynastic effects, whereby genetic risk for physical health conditions in the mother causes increased risk of ADHD in the offspring (19).

## METHODS

### Genetic data: proxies for ADHD and physical health outcomes

Bidirectional MR analyses investigating causal effects used single-nucleotide polymorphisms (SNPs) as proxies for both ADHD (exposure) and physical health (outcomes). For ADHD, 13 SNPs were identified from a GWAS of individuals of European ancestry (19,099 cases and 34,194 control participants) (20) at *P* < 1 ×10^−7^ (Web Table 1) (available at https://doi.org/10.1093/aje/kwaa273). For physical health outcomes, SNPs were identified from GWAS summary statistics of European ancestry populations for cardiometabolic factors (e.g., body mass index (BMI) (21); childhood obesity (22); coronary artery disease (CAD) (23); myocardial infarction (23); hypertension (24); systolic blood pressure (24); type 2 diabetes mellitus (25)), neurological diseases (e.g., migraine (24), epilepsy (26)), autoimmune diseases (e.g., rheumatoid arthritis (27), inflammatory bowel disease (28)), allergic diseases (allergic rhinitis (24), asthma (29), eczema (30)), and lung cancer (31) (details of GWASs on the outcomes listed here can be found in Web Table 2). All outcome GWASs were independent of the ADHD GWAS (20). GWASs for CAD and myocardial infarction were derived from a mixed population sample with 77% White European participants (23). SNPs associated with ADHD were extracted from the respective outcome GWAS (Web Table 3) after removing palindromic sequences. Full details of SNP extraction and quality control are given in Web Appendix 1 and Web Table 3.

**Figure 1:**
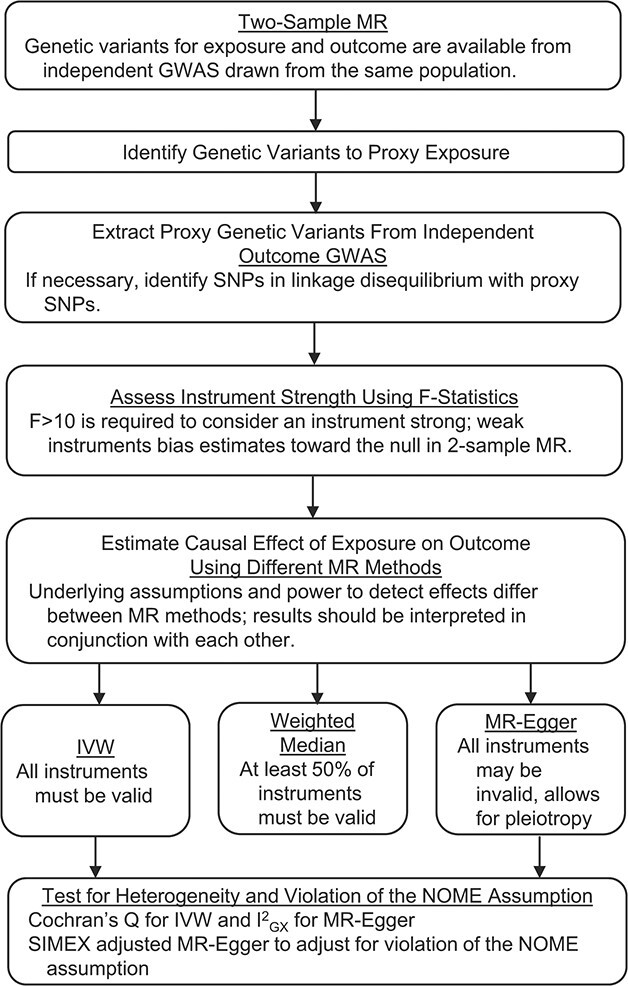
Flow chart of Mendelian randomization (MR) study design outlying MR sensitivity analyses performed, testing for instrument strength and heterogeneity. Abbreviations: GWAS; genome-wide association study; IVW, inverse variance weighted; NOME, no measurement error; SIMEX, simulation extrapolation; SNP, single-nucleotide polymorphism.

**Table 1:** Two-Sample Mendelian Randomization for Attention-Deficit/Hyperactivity Disorder and Physical Health Outcomes, Using Inverse Variant Weighting

**Disease**	**No. of SNPs**	**OR** ^**a**^ ^**b**^	**95% CI**
BMI	10	0.03	−0.01, 0.08
Childhood obesity	9	1.29	1.02, 1.63
Coronary artery disease	11	1.11	1.03, 1.19
Myocardial infarction	11	1.06	0.97, 1.16
Hypertension	11	1.05	0.97, 1.13
Systolic blood pressure	11	−0.01	−0.05, 0.03
Type 2 diabetes mellitus	11	1.09	1.00, 1.20
Migraine	12	0.94	0.84, 1.05
Epilepsy	9	1.01	1.00, 1.02
Rheumatoid arthritis	10	1.02	0.87, 1.19
Inflammatory bowel disease	11	0.99	0.86, 1.15
Allergic rhinitis	11	0.92	0.80, 1.07
Asthma	8	1.16	0.93, 1.45
Eczema	11	0.94	0.82, 1.07
Lung cancer	10	1.10	0.96, 1.27

Abbreviations: BMI, body mass index; CI, confidence interval; OR, odds ratio; SNP, single-nucleotide polymorphism.

^a^ Odds ratios for binary outcomes are to be interpreted as a change in the log odds ratio of the outcome per unit increase in the log odds ratio of ADHD.

^b^ β Values are reported for the continuous outcomes BMI and systolic blood pressure.

### Investigating the casual role of ADHD on physical health outcomes

Two-sample MR was conducted to investigate the causal role of ADHD on physical health outcomes, using GWAS summary statistics to assess both SNP-exposure (i.e., ADHD) and SNP-outcome (i.e., physical health) associations. This allows the estimation of an unconfounded causal effect between exposure and outcome, if certain assumptions (17) hold true: 1) The genetic variants are strongly associated with the exposure of interest; 2) the genetic variants are independent of confounders of the exposure-outcome association; and 3) the genetic variants do not affect the outcome except through the exposure (exclusion restriction criterion). If they affect the outcome through other pathways, this is called horizontal pleiotropy.

The SNP-exposure and SNP-outcome associations were assessed using 3 methods with different assumptions: inverse-variance weighted approach (IVW) (32), weighted median approach (33), and MR-Egger regression (34). The assumptions of different MR methods are summarized in Figure 1.

Odds ratios for associations between binary exposures and binary outcomes in 2-sample MR studies are interpreted as the odds ratio for outcome per unit increase in the log odds ratio of the exposure. When examining binary exposures in MR settings, causal inferences are valid for the continuous liability underlying the binary exposure (35). Thus, when we test for the causal effect of ADHD, we are essentially examining the effect of genetic liability for this exposure, which can be present in an individual even when they do not have an ADHD diagnosis. To avoid repetition, from this point, when we mention causal effects of a binary exposure, such as ADHD, we refer to genetic liability for this exposure.

We present MR *P* values that have not been corrected for multiple testing (while acknowledging the number of correlated phenotypes that have been tested) and focus on consistent results across MR methods to assess the strength of evidence favoring a causal effect.

### Assessing instrument strength, heterogeneity, and outliers

Instrument strength (first MR assumption) was assessed using the *F* statistic (F > 10 suggests results should not suffer from weak instrument bias) (36). Heterogeneity in the MR effect estimates was assessed using Cochran’s Q; when heterogeneity was detected, we performed leave-one-out analysis to detect potential outliers. Heterogeneity in MR-Egger regression was assessed by *I*^2^_GX_ (values < 90% suggest heterogeneity) (37); where heterogeneity was detected, simulation extrapolation adjusted MR-Egger regression was performed (R package SIMEX, version 1.7; R Foundation for Statistical Computing, Vienna, Austria) (37).

### Investigating reverse causation: The causal role of physical health on ADHD

When a potentially causal effect of ADHD on a health outcome was detected, we investigated possible bidirectional effects by repeating the 2-sample MR analyses using independent genetic variants for the relevant physical outcomes as the exposure (childhood obesity, *P* < 1 ×10^−6^ (22); and CAD, *P* < 5 ×10^−8^ (23)) and ADHD as the outcome. Steiger filtering for SNPs was also used to examine whether the instrument SNPs were better predictors of the outcome rather than the exposure, which could indicate reverse causation (38).

### Investigating possible mediators of the association between ADHD and physical health outcomes

Multivariable MR (MVMR) analyses were conducted to assess 2 potential mediators of the association between ADHD and CAD: childhood obesity (22) and lifetime smoking heaviness (39) on CAD. MVMR is an extension of MR that can be used to estimate the causal effects of multiple exposures on 1 outcome simultaneously and requires an additional assumption that each instrument must be conditionally independent of the outcome given all exposures and confounders. MVMR is explained in more detail elsewhere (40).

All steps of the 2-sample MR we performed are summarized in Figure 1. Analyses were conducted using the TwoSampleMR package, version 0.4.14, for R, version 3.4.1.

## RESULTS

### Investigating the causal role of ADHD on physical health outcomes

**Figure 2:**
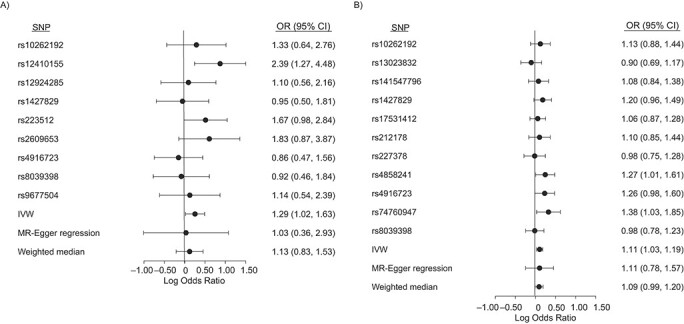
Effect estimates of single variants for attention-deficit hyperactivity disorder on A) childhood obesity and B) coronary artery disease, using inverse variant weighted regression (IVW), weighted median, and MR-Egger approaches. Estimates are shown as odds ratios (ORs) ± 95% confidence intervals (CIs). SNP, single-nucleotide polymorphism.

MR results using the IVW approach are shown in Table 1. There was evidence of a causal effect of ADHD on childhood obesity (odds ratio (OR) =1.29 per log odds increase in ADHD genetic liability, 95% confidence interval (CI): 1.02, 1.63) and CAD (OR = 1.11 per log odds increase in ADHD genetic liability, 95% CI: 1.03, 1.19) using IVW. Both the weighted median estimator and MR-Egger regression showed effects consistently in the same direction as IVW, although with wider confidence intervals, as expected (Figure 2, Web Table 4).

**Table 2:** Bidirectional MR With Causal Estimates for Childhood Obesity and Coronary Artery Disease on Attention-Deficit/Hyperactivity Disorder Using Inverse Variant Weighting, Weighted Medians, and MR-Egger Regression

**Disease**	**No. of SNPs**	**IVW**		**Weighted Median**		**MR-Egger Regression**	
		**OR** ^**a**^	**95% CI**	**OR** ^**a**^	**95% CI**	**OR** ^**a**^	**95% CI**
Childhood obesity	7	1.15	1.05, 1.25	1.10	1.01, 1.20	1.16	0.60, 2.25
Coronary artery disease	37	0.98	0.92, 1.04	0.96	0.88, 1.05	0.91	0.79, 1.06

Abbreviations: CI, confidence interval; IVW, inverse variant weighted; MR, Mendelian randomization; OR, odds ratio; SNP, single-nucleotide polymorphism.

^a^ Odds ratios for binary outcomes should be interpreted as a change in the log odds ratio of the outcome per unit increase in the log odds ratio of the exposure.

There was little evidence of a causal effect of ADHD on BMI, myocardial infarction, hypertension, systolic blood pressure, type 2 diabetes mellitus, migraine, epilepsy, autoimmune and allergic diseases, and lung cancer. There was evidence of a causal effect from MR-Egger regression (OR =1.93, 95% CI, 1.08, 3.45) for inflammatory bowel disease (Web Table 4).

### Assessing heterogeneity

Good instrument strength was indicated by the *F* statistics calculated for all the genetic variants used as instruments (Web Table 5). Heterogeneity in IVW was detected for BMI, hypertension, systolic blood pressure, and allergic rhinitis (Web Table 5). None of the leave-one-out plots identified any outlying SNPs (see Web Figures 1–4). There was no evidence that the MR-Egger intercept differed from the null for any of the heterogeneous tests, suggesting that the detected heterogeneity was unlikely to be due to bias from directional horizontal pleiotropy (Web Table 6).

Based on these initial findings, there was some evidence of a causal effect of ADHD on childhood obesity, CAD, and inflammatory bowel disease. Hence, these 3 outcomes underwent additional sensitivity analyses (Web Appendix 2). Evidence of a causal effect was detected through simulation extrapolation–adjusted MR-Egger regression for childhood obesity and CAD; hence, we also investigated possible bidirectional effects. We did not detect evidence of a causal effect for inflammatory bowel disease through simulation extrapolation adjusted MR-Egger regression (see Web Appendix 2, Web Table 7), which suggested pleiotropic effects; hence, it was not taken forward for further analyses.

### Investigating reverse causation: the casual role of physical health on ADHD

We performed bidirectional MR to examine the effect of CAD and childhood obesity on ADHD (Table 2). There was little evidence of a causal effect of CAD on ADHD with an IVW estimate of 0.98 (95% CI: 0.92, 1.04). However, there was some evidence of a causal effect of childhood obesity on ADHD with an IVW odds ratio of 1.15 (95% CI: 1.05, 1.25) and weighted median odds ratio of 1.10 (95% CI: 1.01, 1.20). MR-Egger regression estimates were directionally consistent and there was little evidence of heterogeneity or horizontal pleiotropy (Web Table 8).

Steiger filtering did not indicate reverse causation, because all the genetic instruments for ADHD explained more variance in ADHD than in CAD or childhood obesity. In addition, none of the 13 SNPs that were used as instruments for ADHD (Web Table 1) were in linkage disequilibrium with any of the 8 SNPs associated with childhood obesity (*P* < 5 ×10^−6^) (22), suggesting there was no overlap between genetic instruments for ADHD and childhood obesity.

### Investigating possible mediators of the association between ADHD and physical health outcomes, using MVMR

**Figure 3:**
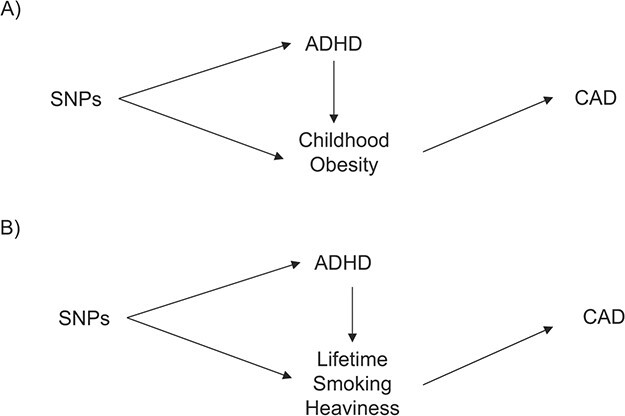
Relationships among A) attention-deficit hyperactivity disorder (ADHD), childhood obesity and coronary artery disease (CAD) and B) among ADHD, childhood obesity, and lifetime smoking heaviness according to results obtained by 3-sample (univariable) Mendelian randomization (MR) and multivariable Mendelian randomization. A) ADHD affects CAD through its effect on childhood obesity, rather than through a direct effect on CAD. B) ADHD affects CAD directly and not only through its effect on lifetime smoking heaviness. Abbreviations: SNP, single nucleotide polymorphism.

Because obesity and smoking are established risk factors for CAD (41) that are strongly associated with ADHD (6, 42), they are possible mediators of the association between ADHD and CAD. When genetic variants for ADHD and childhood obesity were simultaneously entered in the MVMR model, the direct causal effect of ADHD on CAD was attenuated to 1.06 (95% CI: 0.95, 1.17) compared with univariable MR, whereas the effect of childhood obesity on CAD remained stable (OR = 1.14, 95% CI: 1.08, 1.20) (Web Table 9). One explanation for the difference in the potentially causal effects of ADHD on CAD between univariable 2-sample MR and MVMR is that ADHD contributes to CAD through its effect on childhood obesity, rather than through a direct effect on CAD. Thus, there was support for a mediating role of childhood obesity on CAD as illustrated in Figure 3A. The adjusted *F* statistic was >10, indicating good instrument strength (Web Table 9).

The (univariable) causal effect of ADHD on lifetime smoking heaviness was 1.07 (95% CI: 1.04, 1.10). When genetic variants for ADHD and lifetime smoking heaviness were simultaneously entered in the MVMR model, the direct causal effect of ADHD on CAD remained stable (OR = 1.10, 95% CI: 1.00, 1.21), whereas the effect of lifetime smoking heaviness on CAD was attenuated (OR = 1.38, 95% CI: 0.99, 1.92) (Web Table 10). As shown in Figure 3B, this indicates that ADHD potentially affects CAD directly and not only through its effect on lifetime smoking heaviness. However, the adjusted *F* statistic was <10 (Web Table 10), which indicates low instrument strength and which suggested genetic overlap between ADHD and lifetime smoking heaviness; hence, we could not disentangle the effects of ADHD and smoking heaviness on CAD.

## DISCUSSION

In this study, we used a 2-sample MR approach to test putative causal effects of ADHD on physical health outcomes, using genetic variants as instrumental variables as proxies for ADHD. We found evidence consistent with a causal effect of ADHD on CAD and evidence for a bidirectional association between ADHD and childhood obesity. There was little evidence of ADHD causal effects on neurological, autoimmune and allergic diseases, or lung cancer. MVMR results suggested that the causal effect of ADHD on CAD is (at least partially) mediated by childhood obesity.

As reported previously, in observational studies (6, 20, 43), ADHD has been associated with unhealthy lifestyle and risk behaviors. Patients with ADHD are more likely to smoke (42), be overweight (6, 44), and lead a sedentary life (45), and these are all known risk factors for CAD (41). Our results show, indeed, that the effect on CAD was attenuated when we simultaneously assessed the effects of ADHD and childhood obesity on CAD, suggesting childhood obesity may be a potential mediator. Therefore, it seems that at least some of the “impact” of ADHD that confers risk for CAD occurs early in life (i.e., childhood), so if interventions were to target this association, they would need to begin early. We did not identify evidence of causal effect of ADHD on adult BMI, which could indicate that these effects might be more pronounced during childhood when ADHD symptoms are at their peak.

Many studies have shown associations between ADHD and obesity in adolescents and adulthood (44, 46, 47). Motor hyperactivity is a hallmark of ADHD and, therefore, it may appear counterintuitive that patients with ADHD have a higher risk for obesity (48). However, observational studies have shown that those with ADHD have been reported to spend more time watching television (49), have lower levels of physical activity, and increased dysregulation of eating behavior (48).

Bidirectional MR for ADHD and childhood obesity suggested possible effects in both directions, in line with a previous MR study. Maternal effects have been discussed in depth by Martins-Silva et al. (19), who found a causal association of BMI on ADHD using 2-sample MR. Although, typically, ADHD onset is thought to precede childhood obesity, bidirectional analyses could potentially detect dynastic effects, whereby parental genetic risks for physical health conditions cause increased risk of ADHD in the offspring.

When we simultaneously assessed the effects of ADHD and lifetime smoking heaviness on CAD using MVMR, our results suggested an effect of ADHD on CAD independent of lifetime smoking heaviness. However, the adjusted *F* statistic was <10, indicating genetic overlap between ADHD and lifetime smoking heaviness. This is not altogether surprising, because smoking initiation (a core component of the lifetime smoking instrument) is highly correlated with impulsivity and risk taking (39). High genetic correlations between ADHD and smoking initiation have been reported elsewhere (50). Ideally, we would have used smoking heaviness for this analysis, rather than smoking initiation, to better capture the level of tobacco exposure. However, smoking heaviness requires stratification by smoking status, which was not possible using ADHD and CAD summary statistics. Therefore, we emphasize that this exploration of smoking as a possible mediator should be interpreted with caution, because we could not disentangle the effect of ADHD and smoking heaviness on CAD, and should be followed up using smoking heaviness if suitable individual level data become available.

We observed limited evidence supporting a causal role of ADHD on neurological, autoimmune, and allergic diseases. One explanation for this might be that there is no causal effect, and associations found in observational studies are better explained by other factors, such as unmeasured confounding. Another explanation is that these null findings might have arisen due to some of the study limitations, such as instrument validity, population stratification, and low power of MR compared to conventional study designs, which are discussed in more detail in later paragraphs.

### Limitations

The latest ADHD GWAS was the first to identify genetic variants that are significantly associated with ADHD, but these variants still only explain little variation in the ADHD phenotype (20).

We relaxed the *P*-value threshold for SNP inclusion (*P* < 1 ×10^−7^) from the ADHD GWAS to increase the number of instruments so that sensitivity analyses, such as MR-Egger regression, could be performed (3 included SNPs were not genome-wide significant). Because relaxing the threshold for SNP inclusion increases the risk of potential sources of bias, such as pleiotropy or heterogeneity, we deemed increasing the *P* value threshold further would not to be beneficial. Results of our sensitivity analyses suggested there was substantial heterogeneity in MR-Egger regression, which was a marker of measurement error in the instruments. This indicated that there might not have been enough power to detect causal associations, because weak instruments bias associations toward the null in 2-sample MR studies (36). In addition, the age of the participants in the ADHD GWAS would be different from that of some of the outcome GWASs used to extract the instruments. However, genetic instruments are associated with lifetime exposure to a phenotype (in this case, ADHD) even when identified in childhood. ADHD symptoms tend to persist into adulthood in 65% of children diagnosed with the condition (3). Even when ADHD symptoms do not persist into adulthood, childhood ADHD symptoms can potentially have long-lasting effects on physical health later in life (51).

Because of the high correlation between the outcomes tested, we also did not apply a formal correction for multiple testing but focused on consistent results across sensitivity analyses. However, none of our results passed a Bonferroni-corrected multiple testing burden of *P* < 0.0025 (0.05/15), which is likely to be too conservative in this context, and could still possibly be chance findings. Furthermore, although there was little statistical evidence for horizontal pleiotropy, the underlying biological pathways leading to ADHD are unknown for most of the genetic variants and, therefore, the possibility of pleiotropic effects of these variants cannot be discounted. Because in these analyses we used a 2-sample MR framework, which is based on publicly available data, we were not able to test whether the genetic variants used as instruments are independent of potential confounders of the observed exposure to outcome associations. Confounding may also arise due to population stratification (i.e., GWAS sample was not representative of the underlying population or GWAS samples were from mixed populations) (52), assortative mating (i.e., traits are not inherited independently and a consequent violation of the MR assumption that genetic variants are allocated randomly at conception) (53), or selection bias in the GWAS used, all of which might affect both positive and negative findings of our analyses. Furthermore, estimated 2-sample MR odds ratios for associations of binary exposures with binary outcomes can be biased and should only be interpreted in terms of direction and strength of association (35).

In conclusion, using 2-sample MR, we found evidence in favor of a causal effect of ADHD on CAD that is potentially mediated by childhood obesity. Additional research focusing on long-term follow-up of the physical health of children with either a high number of ADHD symptoms or an ADHD diagnosis is required to elucidate these relationships. Our findings strengthen the argument for early and effective treatment of ADHD symptoms in children because the symptoms may also have an impact on later physical health.

## Supplementary Material

Web_Material_kwaa273

## References

[1] Fayyad J , De Graaf R, Kessler R, et al. Cross-national prevalence and correlates of adult attention-deficit hyperactivity disorder. Br J Psychiatry. 2007;190(5):402–409. 17470954 10.1192/bjp.bp.106.034389

[2] Simon V , Czobor P, Bálint S, et al. Prevalence and correlates of adult attention-deficit hyperactivity disorder: meta-analysis. Br J Psychiatry. 2009;194(3):204–211. 19252145 10.1192/bjp.bp.107.048827

[3] Faraone SV , Biederman J, Mick E. The age-dependent decline of attention deficit hyperactivity disorder: a meta-analysis of follow-up studies. Psychol Med. 2006;36(2):159–165. 16420712 10.1017/S003329170500471X

[4] Erskine HE , Norman RE, Ferrari AJ, et al. Long-term outcomes of attention-deficit/hyperactivity disorder and conduct disorder: a systematic review and meta-analysis. J Am Acad Child Adolesc Psychiatry. 2016;55(10):841–850. 27663939 10.1016/j.jaac.2016.06.016

[5] Doshi JA , Hodgkins P, Kahle J, et al. Economic impact of childhood and adult attention-deficit/hyperactivity disorder in the United States. J Am Acad Child Adolesc Psychiatry. 2012;51(10):990–1002.e2. 23021476 10.1016/j.jaac.2012.07.008

[6] Chen Q , Hartman CA, Halkola R-K, et al. Attention-deficit/hyperactivity disorder and clinically diagnosed obesity in adolescence and young adulthood: a register-based study in Sweden. Psychol Med. 2019;49(11):1841–1849. 30220266 10.1017/S0033291718002532 PMC8136973

[7] London AS , Landes SD. Attention deficit hyperactivity disorder and adult mortality. Prev Med. 2016;90:8–10. 27343403 10.1016/j.ypmed.2016.06.021

[8] Fuemmeler BF , Østbye T, Yang C, et al. Association between attention-deficit/hyperactivity disorder symptoms and obesity and hypertension in early adulthood: a population-based study. Int J Obes (Lond). 2011;35(6):852–862. 20975727 10.1038/ijo.2010.214 PMC3391591

[9] Cortese S , Sun S, Zhang J, et al. Association between attention deficit hyperactivity disorder and asthma: a systematic review and meta-analysis and a Swedish population-based study. Lancet Psychiatry. 2018;5(9):717–726. 30054261 10.1016/S2215-0366(18)30224-4

[10] Miyazaki C , Koyama M, Ota E, et al. Allergic diseases in children with attention deficit hyperactivity disorder: a systematic review and meta-analysis. BMC Psychiatry. 2017;17(1):120. 28359274 10.1186/s12888-017-1281-7 PMC5374627

[11] Chen M-H , Su T-P, Chen Y-S, et al. Comorbidity of allergic and autoimmune diseases among patients with ADHD: a nationwide population-based study. J Atten Disord. 2017;21(3):219–227. 23400216 10.1177/1087054712474686

[12] Nielsen PR , Benros ME, Dalsgaard S. Associations between autoimmune diseases and attention-deficit/hyperactivity disorder: a nationwide study. J Am Acad Child Adolesc Psychiatry. 2017;56(3):234–240.e1. 28219489 10.1016/j.jaac.2016.12.010

[13] Instanes JT , Klungsøyr K, Halmøy A, et al. Adult ADHD and comorbid somatic disease: a systematic literature review. J Atten Disord. 2018;22(3):203–228. 27664125 10.1177/1087054716669589 PMC5987989

[14] Hansen TF , Hoeffding LK, Kogelman L, et al. Comorbidity of migraine with ADHD in adults. BMC Neurol. 2018;18(1):147. 30322380 10.1186/s12883-018-1149-6 PMC6190553

[15] Davey Smith G , Ebrahim S. Epidemiology--is it time to call it a day? Int J Epidemiol. 2001;30(1):1–11. 11171840 10.1093/ije/30.1.1

[16] Lawlor DA , Tilling K, Smith GD. Triangulation in aetiological epidemiology. Int J Epidemiol. 2016;45(6):1866–1886. 28108528 10.1093/ije/dyw314 PMC5841843

[17] Davey Smith G , Ebrahim S. “Mendelian randomization”: can genetic epidemiology contribute to understanding environmental determinants of disease? Int J Epidemiol. 2003;32(1):1–22. 12689998 10.1093/ije/dyg070

[18] Davies NM , Holmes MV, Smith GD. Reading Mendelian randomisation studies: a guide, glossary, and checklist for clinicians. BMJ. 2018;362:k601. 30002074 10.1136/bmj.k601 PMC6041728

[19] Martins-Silva T , Dos Santos Vaz J, Hutz MH, et al. Assessing causality in the association between attention-deficit/hyperactivity disorder and obesity: a Mendelian randomization study. Int J Obes (Lond). 2019;43(12):2500–2508. 31000774 10.1038/s41366-019-0346-8

[20] Demontis D , Walters RK, Martin J, et al. Discovery of the first genome-wide significant risk loci for attention deficit/hyperactivity disorder. Nat Genet. 2019;51(1):63–75. 30478444 10.1038/s41588-018-0269-7 PMC6481311

[21] Locke AE , Kahali B, Berndt SI, et al. Genetic studies of body mass index yield new insights for obesity biology. Nature. 2015;518(7538):197–206. 25673413 10.1038/nature14177 PMC4382211

[22] Bradfield JP , Taal HR, Timpson NJ, et al. A genome-wide association meta-analysis identifies new childhood obesity loci. Nat Genet. 2012;44(5):526–531. 22484627 10.1038/ng.2247 PMC3370100

[23] Nikpay M , Goel A, Won H-H, et al. A comprehensive 1,000 genomes-based genome-wide association meta-analysis of coronary artery disease. Nat Genet. 2015;47(10):1121–1130. 26343387 10.1038/ng.3396 PMC4589895

[24] Churchhouse C , Neale B. Rapid GWAS of thousands of phenotypes for 337,000 samples in the UK biobank. Accessed May 4, 2020. http://www.nealelab.is/blog/2017/7/19/rapid-gwas-of-thousands-of-phenotypes-for-337000-samples-in-the-uk-biobank.

[25] Scott RA , Scott LJ, Mägi R, et al. An expanded genome-wide association study of type 2 diabetes in Europeans. Diabetes. 2017;66(11):2888–2902. 28566273 10.2337/db16-1253 PMC5652602

[26] International League Against Epilepsy Consortium on Complex Epilepsies. Genome-wide mega-analysis identifies 16 loci and highlights diverse biological mechanisms in the common epilepsies. Nat Commun. 2018;9(1):5269. 30531953 10.1038/s41467-018-07524-z PMC6288131

[27] Okada Y , Wu D, Trynka G, et al. Genetics of rheumatoid arthritis contributes to biology and drug discovery. Nature. 2014;506(7488):376–381. 24390342 10.1038/nature12873 PMC3944098

[28] Liu JZ , van Sommeren S, Huang H, et al. Association analyses identify 38 susceptibility loci for inflammatory bowel disease and highlight shared genetic risk across populations. Nat Genet. 2015;47(9):979–986. 26192919 10.1038/ng.3359 PMC4881818

[29] Moffatt MF , Gut IG, Demenais F, et al. A large-scale, consortium-based genomewide association study of asthma. N Engl J Med. 2010;363(13):1211–1221. 20860503 10.1056/NEJMoa0906312 PMC4260321

[30] Paternoster L , Standl M, Waage J, et al. Multi-ancestry genome-wide association study of 21,000 cases and 95,000 controls identifies new risk loci for atopic dermatitis. Nat Genet. 2015;47(12):1449–1456. 26482879 10.1038/ng.3424 PMC4753676

[31] Wang Y , McKay JD, Rafnar T, et al. Rare variants of large effect in BRCA2 and CHEK2 affect risk of lung cancer. Nat Genet. 2014;46(7):736–741. 24880342 10.1038/ng.3002 PMC4074058

[32] Lawlor DA , Harbord RM, Sterne JAC, et al. Mendelian randomization: using genes as instruments for making causal inferences in epidemiology. Stat Med. 2008;27(8):1133–1163. 17886233 10.1002/sim.3034

[33] Bowden J , Davey Smith G, Haycock PC, et al. Consistent estimation in Mendelian randomization with some invalid instruments using a weighted median estimator. Genet Epidemiol. 2016;40(4):304–314. 27061298 10.1002/gepi.21965 PMC4849733

[34] Bowden J , Davey Smith G, Burgess S. Mendelian randomization with invalid instruments: effect estimation and bias detection through Egger regression. Int J Epidemiol. 2015;44(2):512–525. 26050253 10.1093/ije/dyv080 PMC4469799

[35] Burgess S , Labrecque JA. Mendelian randomization with a binary exposure variable: interpretation and presentation of causal estimates. Eur J Epidemiol. 2018;33(10):947–952. 30039250 10.1007/s10654-018-0424-6 PMC6153517

[36] Pierce BL , Burgess S. Efficient design for Mendelian randomization studies: subsample and 2-sample instrumental variable estimators. Am J Epidemiol. 2013;178(7):1177–1184. 23863760 10.1093/aje/kwt084 PMC3783091

[37] Bowden J , Del Greco MF, Minelli C, et al. Assessing the suitability of summary data for two-sample Mendelian randomization analyses using MR-Egger regression: the role of the I^2^ statistic. Int J Epidemiol. 2016;45(6):1961–1974. 27616674 10.1093/ije/dyw220 PMC5446088

[38] Hemani G , Tilling K, Smith GD. Orienting the causal relationship between imprecisely measured traits using GWAS summary data. PLoS Genet. 2017;13(11):e1007081. 29149188 10.1371/journal.pgen.1007081 PMC5711033

[39] Wootton RE , Richmond RC, Stuijfzand BG, et al. Evidence for causal effects of lifetime smoking on risk for depression and schizophrenia: a Mendelian randomisation study. Psychol Med. 2020;50(14):2435–2443. 31689377 10.1017/S0033291719002678 PMC7610182

[40] Sanderson E , Davey Smith G, Windmeijer F, et al. An examination of multivariable Mendelian randomization in the single-sample and two-sample summary data settings. Int J Epidemiol. 2019;48(3):713–727. 30535378 10.1093/ije/dyy262 PMC6734942

[41] Khot UN , Khot MB, Bajzer CT, et al. Prevalence of conventional risk factors in patients with coronary heart disease. JAMA. 2003;290(7):898–904. 12928466 10.1001/jama.290.7.898

[42] Lee SS , Humphreys KL, Flory K, et al. Prospective association of childhood attention-deficit/hyperactivity disorder (ADHD) and substance use and abuse/dependence: a meta-analytic review. Clin Psychol Rev. 2011;31(3):328–341. 21382538 10.1016/j.cpr.2011.01.006 PMC3180912

[43] Franke B , Michelini G, Asherson P, et al. Live fast, die young? A review on the developmental trajectories of ADHD across the lifespan. Eur Neuropsychopharmacol. 2018;28(10):1059–1088. 30195575 10.1016/j.euroneuro.2018.08.001 PMC6379245

[44] Nigg JT , Johnstone JM, Musser ED, et al. Attention-deficit/hyperactivity disorder (ADHD) and being overweight/obesity: new data and meta-analysis. Clin Psychol Rev. 2016;43:67–79. 26780581 10.1016/j.cpr.2015.11.005 PMC4800333

[45] Quesada D , Ahmed NU, Fennie KP, et al. A review: associations between attention-deficit/hyperactivity disorder, physical activity, medication use, eating behaviors and obesity in children and adolescents. Arch Psychiatr Nurs. 2018;32(3):495–504. 29784236 10.1016/j.apnu.2018.01.006

[46] Cortese S , Moreira-Maia CR, St. Fleur D, et al. Association between ADHD and obesity: a systematic review and meta-analysis. Am J Psychiatry. 2016;173(1):34–43. 26315982 10.1176/appi.ajp.2015.15020266

[47] Hanć T , Cortese S. Attention deficit/hyperactivity-disorder and obesity: a review and model of current hypotheses explaining their comorbidity. Neurosci Biobehav Rev. 2018;92:16–28. 29772309 10.1016/j.neubiorev.2018.05.017

[48] Khalife N , Kantomaa M, Glover V, et al. Childhood attention-deficit/hyperactivity disorder symptoms are risk factors for obesity and physical inactivity in adolescence. J Am Acad Child Adolesc Psychiatry. 2014;53(4):425–436. 24655652 10.1016/j.jaac.2014.01.009

[49] Lingineni RK , Biswas S, Ahmad N, et al. Factors associated with attention deficit/hyperactivity disorder among US children: results from a national survey. BMC Pediatr. 2012;12:50. 22583686 10.1186/1471-2431-12-50 PMC3502478

[50] Liu M , Jiang Y, Wedow R, et al. Association studies of up to 1.2 million individuals yield new insights into the genetic etiology of tobacco and alcohol use. Nat Genet. 2019;51(2):237–244. 30643251 10.1038/s41588-018-0307-5 PMC6358542

[51] Leppert B , Millard LAC, Riglin L, et al. A cross-disorder PRS-pheWAS of 5 major psychiatric disorders in UK biobank. PLoS Genet. 2020;16(5):e1008185. 32392212 10.1371/journal.pgen.1008185 PMC7274459

[52] Munafò M , Smith GD. Biased estimates in Mendelian randomization studies conducted in unrepresentative samples. JAMA Cardiol. 2018;3(2):181. 10.1001/jamacardio.2017.4279 29238819

[53] Hartwig FP , Davies NM, Davey SG. Bias in Mendelian randomization due to assortative mating. Genet Epidemiol. 2018;42(7):608–620. 29971821 10.1002/gepi.22138 PMC6221130

