# Supplementary material for: The Effect of Attention Deficit/Hyperactivity Disorder on Physical Health Outcomes: A 2-Sample Mendelian Randomization Study
Source: Am J Epidemiol. 2020 Dec 16;190(6):1047–55. doi: 10.1093/aje/kwaa273 (PMC8168225; doi:10.1093/aje/kwaa273)
Supplement: Web_Material_kwaa273 [file web_material_kwaa273.pdf]

Web Material for

The Effect of ADHD on Physical Health Outcomes

# - A Two-Sample Mendelian Randomization Study

Running head: Mendelian Randomization of ADHD and Physical Health

Web Appendices 1–2

Web Tables 1–10

Web Figures 1–4

Web Appendix 1. Details on SNP extraction and quality control

Genome-wide association studies (GWAS) derived from UK Biobank by the Neale lab (1) are reported on a risk difference scale and were transformed onto the log OR scale.

Approximate transformation of association estimates for binary traits in UK Biobank GWAS from the risk difference scale to the log odds scale:

log_OR = beta/mu(1-mu)

se_log_OR = se/mu(1-mu)

where mu=n_cases/n_total.

If SNPs associated with ADHD could not be extracted from the outcome GWAS (due to differences in genotyping chips, reference panels or imputation methods between studies), we searched for proxies using the Single Nucleotide Polymorphisms Annotator (SniPA) with r2=0.9 (https://snipa.helmholtz-muenchen.de/snipa3/) (Munich, Germany) (2).

Web Appendix 2. Additional sensitivity analyses for childhood obesity, coronary artery disease (CAD) and inflammatory bowel disease (IBD)

I^2_{GX} estimates for childhood obesity (I^2_{GX}=47%), CAD (I^2_{GX}=48%) and IBD (I^2_{GX}=48%) (see Web Table 5 for all I^2_{GX} estimates) were low. Hence, we performed Simulation Extrapolation (SIMEX) adjusted MR-Egger regression to adjust MR-Egger estimates accordingly (Web Table 7). The SIMEX point estimate for CAD was consistent with the original IVW and MR-Egger estimate (OR:1.11 (95% CI:1.03,1.20). For childhood obesity the MR SIMEX estimate (OR:1.26 (95% CI:0.99,1.60) was increased in comparison to the MR-Egger estimate and was closer to the IVW estimate of 1.29. The effect of genetic liability for ADHD on IBD in MR-Egger regression was completely attenuated with OR of 1.00 (95% CI:0.86,1.17) after SIMEX

adjustment, which is in line with the IVW estimate of 0.99 and hence was not taken forward for further analyses.

Web Table 1: ADHD genetic variants associated at $p<1x10^{-7}$(3)

	SNP	effect allele	other allele	beta	SE	p-value
1	rs17531412	A	G	0.105	0.015	$1.1x10^{-12}$
2	rs1427829	A	G	0.082	0.014	$1.3x10^{-9}$
3	rs8039398	T	C	-0.080	0.014	$3.0x10^{-9}$
4	rs4858241	T	G	0.082	0.014	$8.2x10^{-9}$
5	rs28411770	T	C	0.086	0.015	$1.2x10^{-8}$
6	rs212178	A	G	-0.117	0.021	$1.2x10^{-8}$
7	rs74760947	A	G	-0.180	0.032	$1.4x10^{-8}$
8	rs4916723	A	C	-0.078	0.014	$1.8x10^{-8}$
9	rs1222063	A	G	0.096	0.017	$3.1x10^{-8}$
10	rs10262192	A	G	0.074	0.014	$3.7x10^{-8}$
11	rs227378	A	C	0.079	0.015	$6.5x10^{-8}$
12	rs13023832	A	G	0.115	0.022	$9.3x10^{-8}$
13	rs141547796	A	G	-0.137	0.026	$9.6x10^{-8}$

Abbreviations: standard error, SE.

Web Table 2. Details of GWAS used to identify instruments for outcomes

	GWAS references	*Year*	*Ancestry*	*Cohort/Consortium*	N_{case}	$N_{control}$
Cardiometabolic diseases						
BMI	(4)	2015	European	GIANT	-	322,154
Childhood obesity	(5)	2012	European	EGG	5,530	8,318
Coronary artery disease	(6)	2015	Mixed	CARDIoGRAMplusC4D	60,801	123,504
Myocardial infarction	(6)	2015	Mixed	CARDIoGRAMplusC4D	43,676	128,199
Hypertension	(1)	2017	European	UKBB	87,690	249,469
Systolic blood pressure	(1)	2017	European	UKBB	-	317,754
Type II diabetes mellitus	(7)	2017	European	18 studies[a]	26,676	132,532
Neurological diseases						
Migraine	(1)	2017	European	UKBB	10,007	327,152
Epilepsy	(8)	2018	European	ILAE	15,212	29,677
Immunological diseases						
Rheumatoid arthritis	(9)	2014	European	18 studies[b]	14,361	43,923
Inflammatory bowel disease	(10)	2015	European	IIBDGC	12,882	21,770
Allergic rhinitis	(1)	2017	European	UKBB	18,934	64,595
Asthma	(11)	2010	European	GABRIEL	10,365	16,110
Eczema	(12)	2015	European	EAGLE	10,788	30,047
Cancer						
Lung Cancer	(13)	2014	European	ILCCO	11,348	15,861

Abbreviations: Genome Wide Association Study, GWAS; Body mass index, BMI; Genetic Investigation of ANthropometric Traits, GIANT; Early Growth Genetics Consortium, EGG; Coronary ARtery DIsease Genome wide Replication and Meta-analysis (CARDIoGRAM) plus The Coronary Artery Disease (C4D) Genetics, CARDIoGRAMplusC4D; UK Biobank, UKBB; International League Against Epilepsy, ILAE; International Inflammatory Bowel Disease Genetics Consortium, IIBDGC; Consortium Large-Scale Genome-Wide Association Study of Asthma, GABRIEL; EArly Genetics and Lifecourse Epidemiology, EAGLE; International Lung Cancer Consortium, ILCCO.

[a]ARIC, BioMe, deCODE, DGDG, DGI, EGCUT-370, EGCUT-OMNI, EPIC-InterAct, FHS, FUSION, GoDARTS, HPFS, KORAgen, NHS, PIVUS, RS-I, ULSAM, WTCCC

[b]BRASS, CANADA, EIRA, NARAC1, NARAC2, WTCCC, Rheumatoid Arthritis Consortium International for Immunochip (RACI)-UK, RACI-US, RACI-SE-E, RACI-SE-U, RACI-NL, RACI-ES, RACI-i2b2, ReAct, Dutch (including AMC, BeSt, LUMC and DREAM), anti-TNF response to therapy collection (ACR-REF: BRAGGSS, BRAGGSS2, ERA, KI and TEAR), CORRONA, Vanderbilt

Web Table 3: Genetic variants included in two-sample MR analyses.

	independent SNPs associated with ADHD at $p<1x10^{-7}$													
	rs10262192	rs1222063	rs13023832	rs141547796	rs1427829	rs17531412	rs212178	rs227378	rs28411770	rs4858241	rs4916723	rs74760947	rs8039398	rs11591402
Cardiometabolic diseases														
BMI			rs9677504			rs12410155	rs12924285	rs223512				rs2609653		
Coronary Heart Disease														
Childhood obesity			rs9677504			rs12410155	rs12924285	rs223512				rs2609653		
Myocardial infarction														
Hypertension			rs9677504					rs150900						
Type II Diabetes mellitus														
Systolic Blood pressure			rs9677504					rs150900						
Neurological diseases														
Migraine			rs9677504					rs150900						rs6584649
Epilepsy			rs9677504											
Autoimmune and Allergic Diseases														
Rheumatoid Arthritis				rs56068671								rs2609653		
Inflammatory Bowel Disease														
Allergic rhinitis			rs9677504					rs150900						
Asthma	rs2106900		rs1912185		rs704061	rs12410155		rs223504					rs281320	
Eczema														

Abbreviations: Body mass index, BMI.

Green = SNP extracted, Yellow = proxy SNP extracted, Black = SNP not available, Red = SNP palindromic and excluded.

Web Table 4: Two-sample MR for ADHD and physical health outcomes using weighted median and MR-Egger regression

**Disease**	**N of SNPs**	**Weighted median**			**MR-Egger**		
		OR[a,b]	*95%CI*	*p-value*	*OR*[a,b]	*95%CI*	*p-value*
BMI	10	0.00	-0.05,-0.04	0.832	-0.07	0.28,0.14	0.541
Childhood obesity	9	1.13	0.82,1.56	0.444	1.03	0.36,2.93	0.919
Coronary artery disease	11	1.09	0.99,1.20	0.067	1.11	0.78,1.57	0.584
Myocardial infarction	11	1.05	0.94,1.16	0.424	1.11	0.71,1.73	0.654
Hypertension	11	1.04	0.97,1.11	0.247	0.95	0.66,1.37	0.793
Systolic blood pressure	11	-0.01	-0.04,0.03	0.746	-0.04	-0.21,-0.13	0.637
Type II diabetes mellitus	11	1.11	0.98,1.25	0.102	0.92	0.61,1.37	0.677
Migraine	12	0.89	0.76,1.03	0.111	0.64	0.40,1.04	0.099
Epilepsy	9	1.01	0.99,1,03	0.181	1.00	0.95,1.07	0.906
Rheumatoid arthritis	10	1.10	0.88,1.36	0.405	1.00	0.47,2.12	0.997
Inflammatory bowel disease	11	0.96	0.81,1.14	0.654	1.93	1.08,3.45	0.052
Allergic rhinitis	11	0.86	0.75,0.99	0.038	1.10	0.57,2.13	0.783
Asthma	8	1.13	0.88,1.46	0.331	0.64	0.14,2.97	0.591
Eczema	11	0.98	0.82,1.1	0.835	0.79	0.43,1.46	0.474
Lung Cancer	10	1.08	0.89,1.31	0.436	1.04	0.56,1.94	0.899

Abbreviations: Mendelian randomization, MR; Single nucleotide polymorphisms, SNPs; Body mass index, BMI; Odds ratio, OR; Confidence interval, CI.

[a] Odds ratios for binary outcomes are to be interpreted as a change in the log odds ratio of the outcome per unit increase in the log odds ratio of ADHD.

[b] Values are expressed as betas for the continuous outcomes BMI and systolic blood pressure.

Web Table 5: Measures of heterogeneity and instrument strength in MR approaches.

**Disease**	**F-statistic** SNP-ADHD[a]	**Cochranes Q** p-value[b]	**I^2_{GX}**
Cardiometabolic diseases			
BMI	33.87	0.006	0.42
Childhood obesity	33.96	0.318	0.47
Coronary artery disease	33.46	0.472	0.48
Myocardial infarction	33.46	0.234	0.48
Hypertension	33.45	$8x10^{-5}$	0.48
Systolic blood pressure	33.95	$2x10^{-5}$	0.5
Type II diabetes mellitus	33.46	0.934	0.48
Neurological diseases			
Migraine	33.3	0.385	0.43
Epilepsy	34.49	0.80	0.48
Autoimmune and Allergic Diseases			
Rheumatoid arthritis	33.72	0.362	0.5
Inflammatory bowel disease	33.46	0.265	0.48
Allergic rhinitis	33.45	0.003	0.48
Asthma	33.61	0.151	0
Eczema	33.46	0.478	0.48
Cancers			
Lung Cancer	33.89	0.576	0.52

Abbreviations: Mendelian randomization, MR; Single nucleotide polymorphisms, SNPs; Body mass index, BMI; Odds ratio, OR; Confidence interval, CI.

[a] Variants associated with ADHD that could be extracted from the corresponding disease GWAS summary statistics

[b] p value for IVW

Web Table 6: Pleiotropy in MR-Egger regression assessed by the MR-Egger intercept

**Disease**	**SNPs**	**MR-Egger intercept**		
		OR	*95%CI*	*p-value*
Cardiometabolic diseases				
BMI	10	1.01	0.99,1.03	0.354
Childhood obesity	9	1.02	0.92,1.12	0.717
Coronary artery disease	11	1.00	0.97,1.03	0.994
Myocardial infarction	11	1.00	0.96,1.04	0.848
Hypertension	11	1.00	1.00,1.01	0.604
Systolic blood pressure	11	1.00	0.99,1.02	0.722
Type II diabetes mellitus	11	1.02	0.98,1.06	0.396
Neurological diseases				
Migraine	12	1.00	1.00,1.00	0.140
Epilepsy	9	1.00	1.00,1.01	0.848
Autoimmune and Allergic Diseases				
Rheumatoid arthritis	10	1.00	0.93,1.08	0.965
Inflammatory bowel disease	11	0.94	0.89,0.99	0.046
Allergic rhinitis	11	1.00	0.99,1.01	0.606
Asthma	8	1.05	0.92,1.20	0.473
Eczema	11	1.02	0.96,1.07	0.594
Cancer				
Lung Cancer	10	1.01	0.95,1.07	0.856

Abbreviations: Mendelian randomization, MR; Single nucleotide polymorphisms, SNPs; Body mass index, BMI; Odds ratio, OR; Confidence interval, CI.

Web Table 7: Causal estimates for ADHD on physical diseases using MR-Egger and SIMEX adjusted MR-Egger regression.

**Disease**	**SNPs**	**MR-Egger**				**SIMEX adjusted MR-Egger**			
		**slope**		**intercept**		**slope**		**intercept**	
		OR(95%CI)	*p-value*	*OR(95%CI)*	*p-value*	*OR(95%CI)*	*p-value*	*OR(95%CI)*	*p-value*
Childhood obesity	9	1.06(0.37,3.07)	0.919	1.02(0.92,1.12)	0.717	1.26(0.99,1.60)	0.102	1.01(0.99,1.04)	0.258
Coronary Heart disease	11	1.11(0.78,1.57)	0.584	1.00(0.97,1.03)	0.994	1.11(1.03,1.20)	0.028	1.00(0.99,1.01)	0.852
Inflammatory bowel disease	11	1.93(1.08,3.45)	0.052	0.94(0.89,0.99)	0.046	1.00(0.86,1.17)	0.992	1.00(0.98,1.01)	0.713

Abbreviations: Simulation Extrapolation, SIMEX; Mendelian randomization, MR; Single nucleotide polymorphisms, SNPs; Odds ratio, OR; Confidence interval, CI.

Web Table 8: Pleiotropy in bidirectional MR assessed by the MR-Egger intercept

**Disease**	**SNPs**	**MR-Egger intercept**		
		OR	*95%CI*	*p-value*
Childhood obesity	7	1.00	0.88,1.13	0.972
Coronary artery disease	37	1.01	0.99,1.02	0.313

Abbreviations: Mendelian randomization, MR; Single nucleotide polymorphisms, SNPs; Odds ratio, OR; Confidence interval, CI.

Web Table 9: Multivariable MR for ADHD on coronary artery disease with genetic liability for childhood obesity as covariate.

	IVW									
	**Univariable**				**Multivariable**					
	**SNP**	*OR*	*95% CI*	*p-value*	*SNP*	*OR*	*95% CI*	*p-value*	*F*	*Q (p)*
ADHD	9	1.10	1.01,1.19	0.022	16	1.06	0.95,1.17	0.310	14.61	18 (0.14)
Childhood obesity	7	1.15	1.08,1.23	$9.2x10^{-6}$		1.14	1.08,1.20	$2.5x10^{-4}$	14.50	

Abbreviations: Inverse Variant Weighted, IVW; Mendelian randomization, MR; Single nucleotide polymorphisms, SNPs; Odds ratio, OR; Confidence interval, CI.

Web Table 10: Multivariable MR for ADHD on coronary artery disease with genetic liability for lifetime smoking as covariate.

	IVW									
	**Univariable**				**Multivariable**					
	SNP	*OR*	*95% CI*	*p-value*	*SNP*	*OR*	*95% CI*	*p-value*	*F*	*Q (p)*
ADHD	9	1.19	1.09,1.29	$6.1x10^{-5}$	134	1.10	1.00,1.21	0.045	2.91	237 (<0.001)
Lifetime smoking	130	1.69	1.30,2.19	$8.6x10^{-5}$		1.38	0.99,1.92	0.061	7.96	

Abbreviations: Inverse Variant Weighted, IVW; Mendelian randomization, MR; Single nucleotide polymorphisms, SNPs; Odds ratio, OR; Confidence interval, CI.

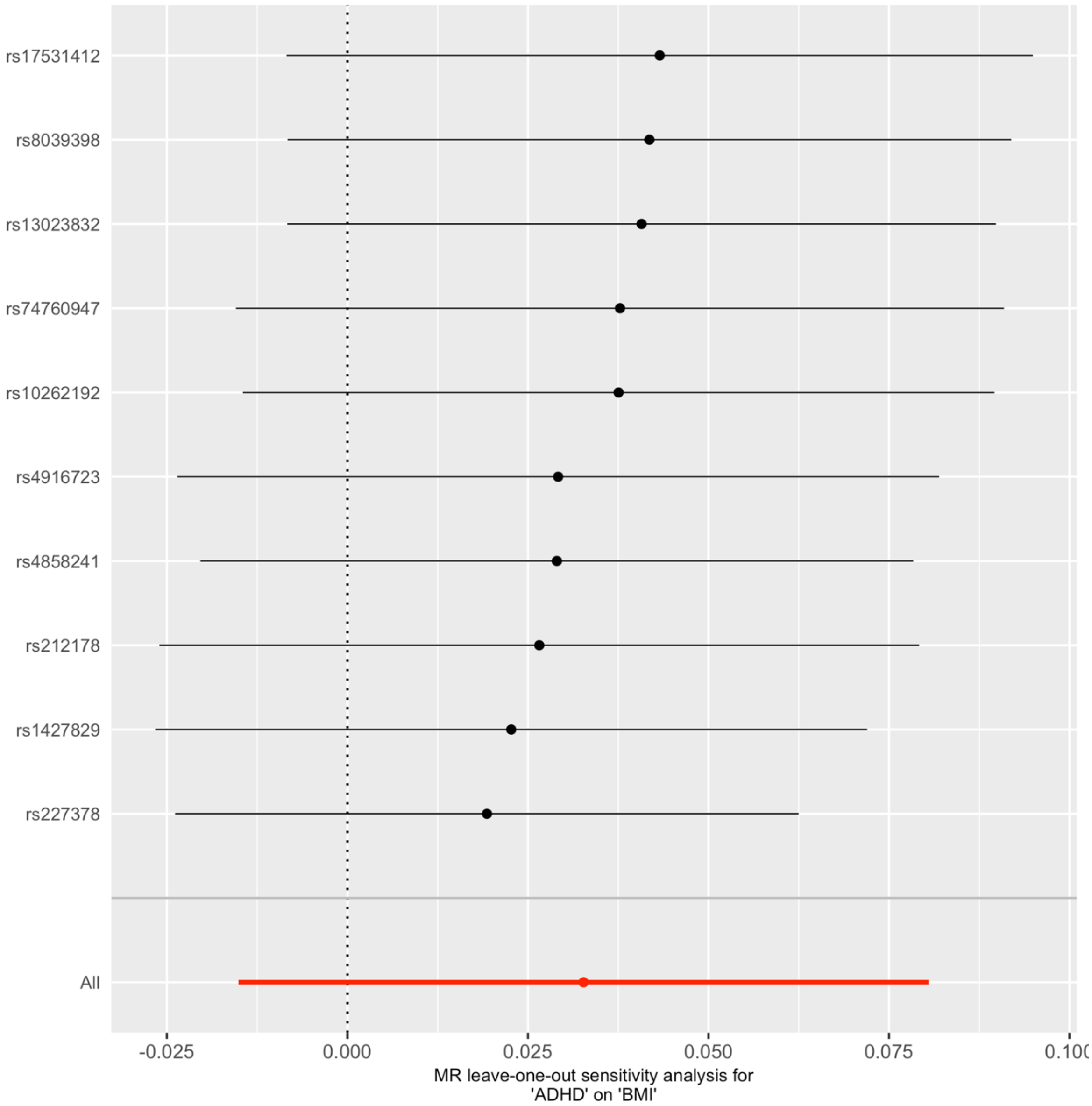

Web Figure 1: Leave-one out sensitivity analysis for IVW MR estimate of ADHD on BMI.

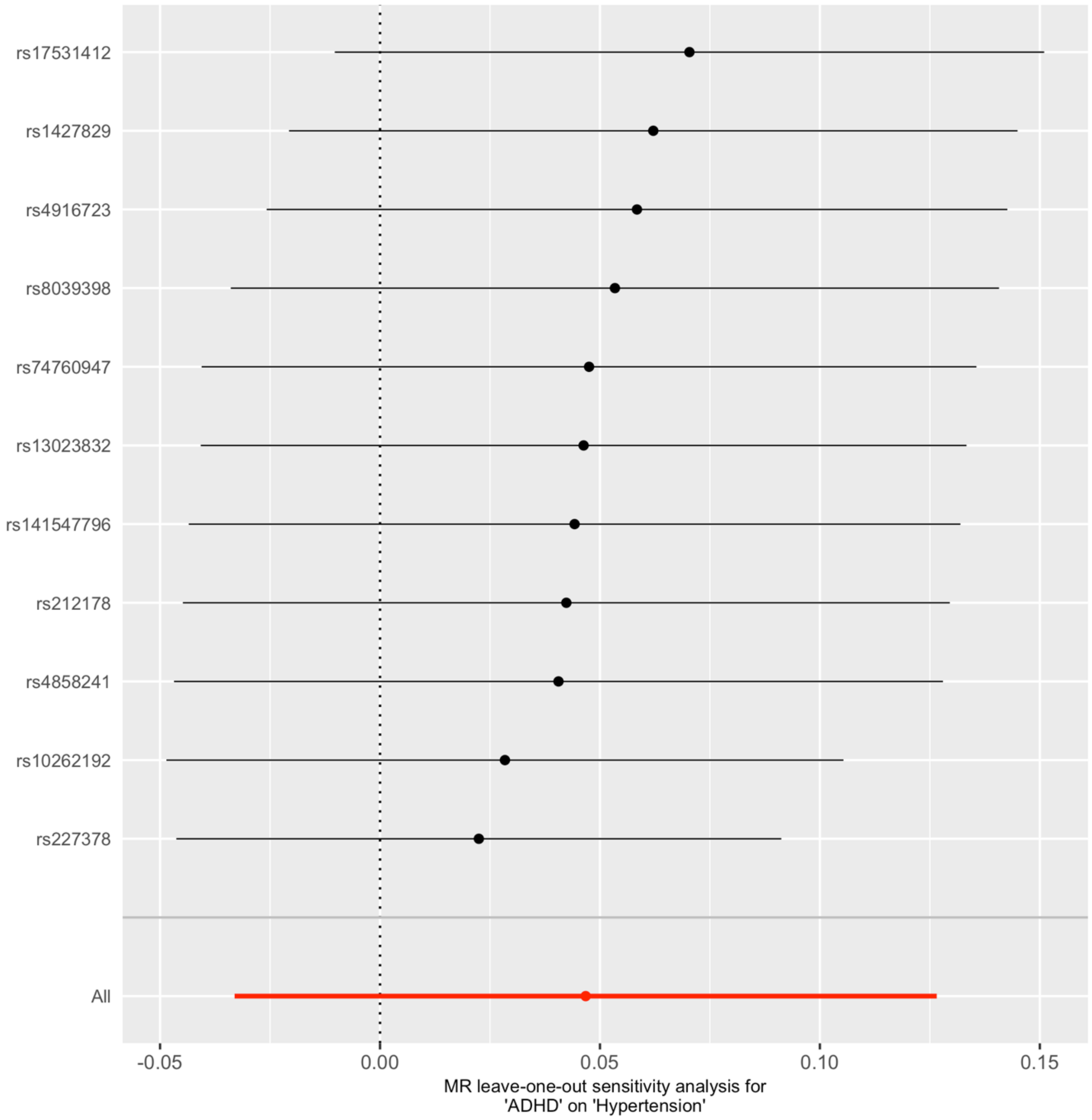

Web Figure 2: Leave-one out sensitivity analysis for IVW MR estimate of ADHD on hypertension.

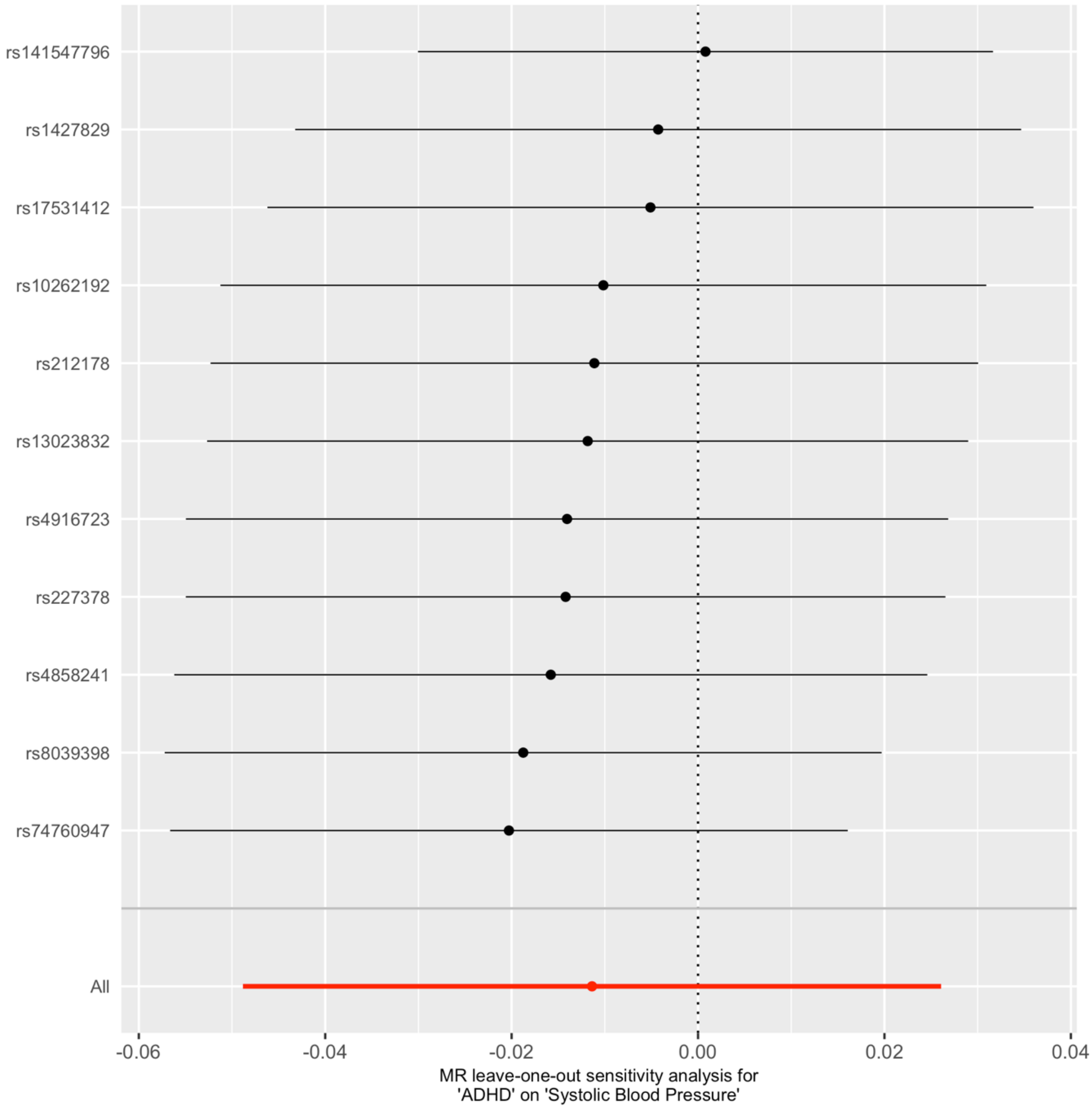

Web Figure 3: Leave-one out sensitivity analysis for IVW MR estimate of ADHD on systolic blood pressure.

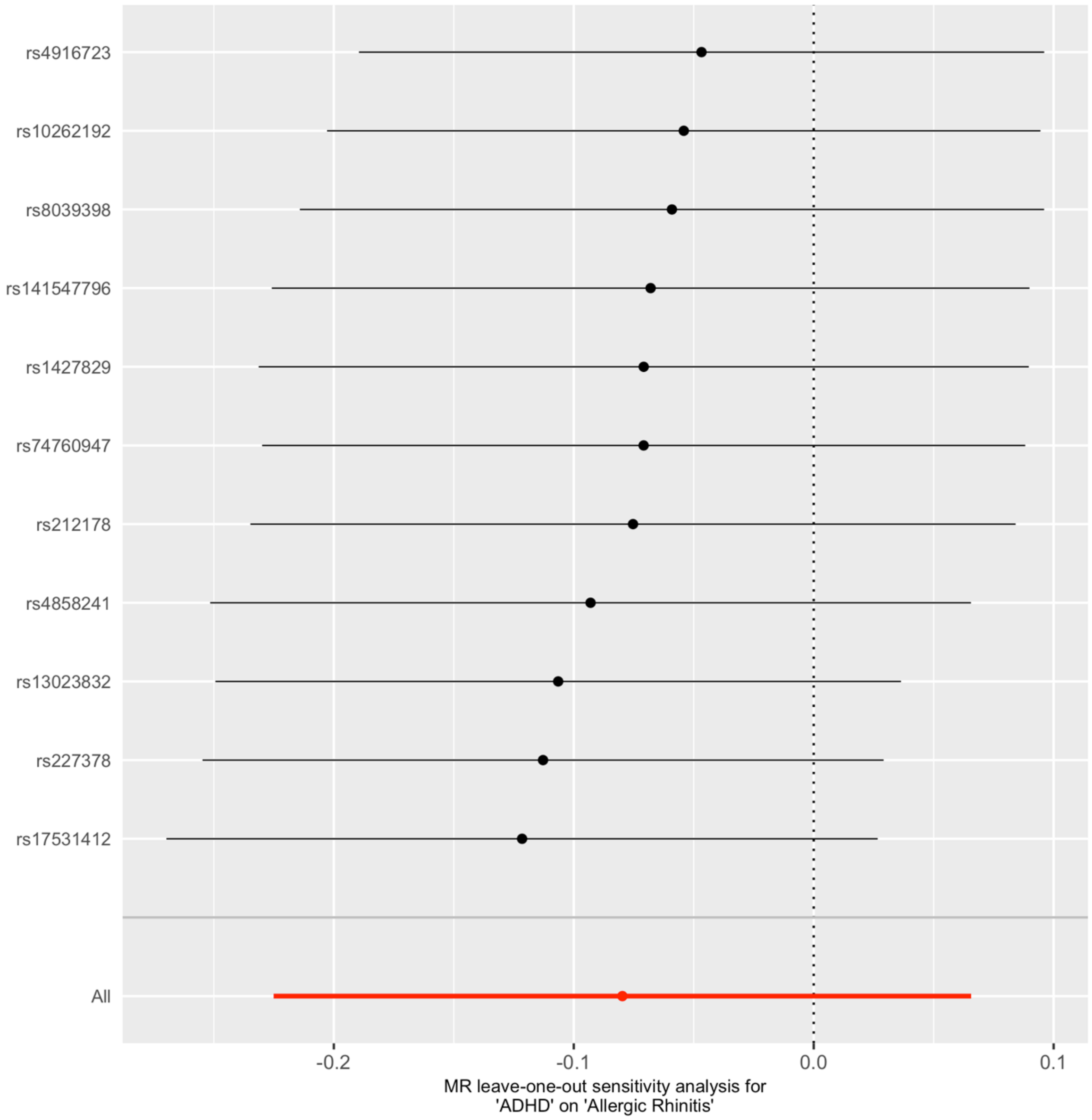

Web Figure 4: Leave-one out sensitivity analysis for IVW MR estimate of ADHD on allergic rhinitis.

References

1. Churchhouse C, Neale B. Rapid GWAS of thousands of phenotypes for 337,000 samples in the UK Biobank. *Neale lab*. (http://www.nealelab.is/blog/2017/7/19/rapid-gwas-of-thousands-of-phenotypes-for-337000-samples-in-the-uk-biobank). (Accessed May 4, 2020)

2. Arnold M‡, Raffler J‡, Pfeufer A, et al. SNiPA: an interactive, genetic variant-centered annotation browser. Bioinformatics (2014). Available at http://www.snipa.org. (Accessed May 4, 2020).

3. Demontis D, Walters RK, Martin J, et al. Discovery of the first genome-wide significant risk loci for attention deficit/hyperactivity disorder. *Nat. Genet.* 2019;51(1):63–75.

4. Locke AE, Kahali B, Berndt SI, et al. Genetic studies of body mass index yield new insights for obesity biology. Nature. 2015;518(7538):197–206.

5. Bradfield JP, Taal HR, Timpson NJ, et al. A genome-wide association meta-analysis identifies new childhood obesity loci. Nat. Genet. 2012;44(5):526–531.

6. Nikpay M, Goel A, Won H-H, et al. A comprehensive 1,000 Genomes-based genome-wide association meta-analysis of coronary artery disease. *Nat. Genet.* 2015;47(10):1121–1130.

7. Scott RA, Scott LJ, Mägi R, et al. An Expanded Genome-Wide Association Study of Type 2 Diabetes in Europeans. Diabetes. 2017;66(11):2888–2902.

8. International League Against Epilepsy Consortium on Complex Epilepsies. Genome-wide mega-analysis identifies 16 loci and highlights diverse biological mechanisms in the common epilepsies. Nat. Commun. 2018;9(1):5269.

9. Okada Y, Wu D, Trynka G, et al. Genetics of rheumatoid arthritis contributes to biology and drug discovery. Nature. 2014;506(7488):376–381.

10. Liu JZ, van Sommeren S, Huang H, et al. Association analyses identify 38 susceptibility loci for inflammatory bowel disease and highlight shared genetic risk across populations. Nat. Genet. 2015;47(9):979–986.

11. Moffatt MF, Gut IG, Demenais F, et al. A large-scale, consortium-based genomewide association study of asthma. N. Engl. J. Med. 2010;363(13):1211–1221.

12. Paternoster L, Standl M, Waage J, et al. Multi-ancestry genome-wide association study of 21,000 cases and 95,000 controls identifies new risk loci for atopic dermatitis. Nat. Genet. 2015;47(12):1449–1456.

13. Wang Y, McKay JD, Rafnar T, et al. Rare variants of large effect in BRCA2 and CHEK2 affect risk of lung cancer. Nat. Genet. 2014;46(7):736–741.
